# No Socioeconomic Inequalities in Mortality among Catholic Monks: A Quasi-Experiment Providing Evidence for the Fundamental Cause Theory

**DOI:** 10.1177/00221465241291847

**Published:** 2024-11-14

**Authors:** Alina Schmitz, Patrick Lazarevič, Marc Luy

**Affiliations:** 1 TU Dortmund University, Dortmund, Germany; 2 Statistics Austria, Wien, Austria; 3 Vienna Institute of Demography of the Austrian Academy of Sciences, Wien, Austria

**Keywords:** cloister study, health inequalities, mortality, religious communities, socioeconomic status

## Abstract

We propose a novel approach to test the fundamental cause theory (FCT) by analyzing the association between socioeconomic status (SES), as measured by the order titles “brothers” and “padres,” and mortality in 2,421 German Catholic monks born between 1840 and 1959. This quasi-experiment allows us to study the effect of SES on mortality in a population with largely standardized living conditions. Mortality analyses based on Kaplan-Meier product limit estimation show that there were no statistically significant survival differences between the high and lower SES monks. This holds for all birth cohorts, indicating that monastic life offers health protection for monks with a lower SES regardless the disease patterns, causes of death, or main risk factors in a given period. These findings support the FCT: Whereas SES-related differences in mortality are a widely confirmed finding in the general population, a context with largely standardized conditions eliminates the importance of SES-related resources.

Numerous studies document socioeconomic inequalities in health and longevity: People with a lower socioeconomic status (SES), which is typically measured by education, income, or occupational position, are at higher risk of poor health and mortality than their counterparts from higher SES groups (see the overviews in Bosworth 2018; Marmot 2005). These health inequalities are substantial, typically amounting to a 5- to 10-year difference in average life expectancy at birth and a 10- to 20-year difference in disability-free life expectancy (Mackenbach 2012:761). Poor health and a lower life expectancy are not only found in the lowest SES groups (e.g., among those at risk of poverty or the low educated) but also affect those who are relatively well off compared to even higher SES groups (Marmot 2004; Winkler-Dworak 2008). This is why it is also referred to as the “social gradient in health” (Adler et al. 1994).

Although the association between SES and mortality does not appear to be linear and the magnitude of the social gradient changes over time (Everett, Rehkopf, and Rogers 2013; Montez, Hummer, and Hayward 2012), SES inequalities in mortality are consistently observed and persist across time and place (Balaj et al. 2024; Gupta and Sudharsanan 2022; Long et al. 2023; Mackenbach et al. 2008; Mackenbach, Kulhánová, Bopp, et al. 2015; Murtin et al. 2021; Sasson and Hayward 2019). The persistence of the social gradient is particularly striking given that diseases and risk factors that were the leading causes of death in earlier periods (notably infectious diseases, such as diphtheria, measles, typhoid fever, and tuberculosis) have been eradicated in high-income countries. Rather than disappearing, however, SES inequalities in mortality are reflected in today’s leading causes of death, including cancer and cardiovascular disease (Phelan, Link, and Tehranifar 2010:S28).

There are several explanations for the social gradient in health and mortality (for a detailed literature review, see Mackenbach 2012). In the following, we provide a brief overview of explanations that focus on specific groups of risk factors before focusing on the fundamental cause theory (FCT), proposed by Link and Phelan (1995).

## Background

### Socioeconomic Inequalities in Health and Mortality: Explanations for the Social Gradient

In general, explanatory approaches can be distinguished by the causal relationship they assume between SES and health: SES affects health (social causation), health affects SES (health selection), and common (unknown) underlying factors, such as genetic endowment, cognitive abilities, or personality, affect both health and SES (indirect selection; Kröger, Pakpahan, and Hoffmann 2015). Although the latter is particularly difficult to assess empirically, studies suggest that both health selection and social causation play a role. However, as Hoffmann, Kröger, and Pakpahan (2018) show, their relative importance appears to change over the life course. At older ages (the phase of life when the risk of morbidity and mortality rises sharply), health selection processes decline, making social causation the dominant mechanism driving health inequalities in later life.

With regard to the specific factors underlying social causation, the explanatory approaches emphasize different aspects. However, they share a basic assumption: Whereas higher SES groups generally have good access to health-relevant resources (e.g., education and the associated human capital, various psychosocial resources) and are less affected by health-damaging factors, lower SES groups have rather limited resources and are more exposed to health-damaging factors, which can also accumulate over time and ultimately lead to poor health compared to higher SES groups (for a detailed review, see Mackenbach 2012).

In this sense, (neo-)materialist explanations highlight the importance of material factors, such as financial means, working and housing conditions, and access to goods, services, and health care (e.g., Granström et al. 2015; Lynch et al. 2000). Psychosocial explanations emphasize the unequal distribution of risk factors, such as high levels of stress in daily life, social conflict, lack of social support, and social exclusion (e.g., Link et al. 2024; Marmot and Wilkinson 2001; Soskolne and Manor 2010). Behavioral explanations suggest that health inequalities result from the higher prevalence of smoking, excessive alcohol consumption, physical inactivity, and inadequate nutrition in lower SES groups (e.g., Kershaw et al. 2013; Kraft and Kraft 2021; Pampel, Krueger, and Denney 2010). The relative contribution of these three groups of explanatory factors is debated. However, studies suggest that material deprivation has not only direct negative effects on health but also indirect effects (Moor, Spallek, and Richter 2017; Schmitz and Pförtner 2018), such as smoking being used to reduce the stress of facing financial hardship in groups with lower SES (Pampel et al. 2010).

Finally, and importantly, explanations of cultural capital postulate that SES is strongly linked to habitus, that is, typical ways of interpreting situations and acting accordingly, which, in turn, strongly determines influences (e.g., Bourdieu 1984). Cockerham (2005) builds on this idea by emphasizing the interplay between social structure and individual agency in producing health outcomes in his health lifestyle theory (for an update and extension with a focus on changes induced by the COVID-19 pandemic and digitalization, see also Cockerham 2023). This theory posits that structural variables (i.e., class circumstances and living conditions) on the one hand create life chances (structure), which then enable or constrain certain life choices (agency), and on the other hand, through experience and socialization, result in preferences for certain life choices over others, which, in turn, affect life chances. This interplay between structure and agency produces a habitus, that is, an internalized tendency to evaluate situations in a certain way and to act accordingly. Replication of these behaviors ultimately produces health lifestyles and health outcomes. Freese and Lutfey (2011) recognize the habitus associated with SES as an important metamechanism for producing systematic health outcomes by influencing preferences for more or less harmful health behaviors.

### The Fundamental Cause Theory

Rather than focusing on specific (groups of) risk factors or health-promoting factors, Link and Phelan (1995) proposed their fundamental cause theory (FCT) to explain the persistence of health inequalities across time and place. Link and Phelan (1995:80) argue that rather than focusing on relatively proximal causes of disease, including health behaviors, “individually based risk factors must be contextualized, by examining what puts people at risk.” The FCT postulates that social factors, first and foremost a person’s SES, are the fundamental determinants of health because their “health effects cannot be eliminated by addressing the mechanisms that appear to link them to disease” (Link and Phelan 1995:86). Accordingly, a high SES is associated with access to multiple and flexibly usable resources, such as money, knowledge, prestige, power, and beneficial social connections, which can be used to avoid disease risks and/or to minimize the consequences of disease (Phelan et al. 2004:265). Thus, the health-protective effect of a high SES is independent of the current epidemiological disease profile and the relevant risk factors. The main argument for this is that as long as data are available, there always was a social gradient of health, regardless of which diseases (infectious diseases or chronic diseases, including today’s leading causes of death) are currently relevant.

Accordingly, the specific risk factors that mediate the link between SES and health are constantly changing, whereas the underlying fundamental causes, that is, differences in individuals’ access to resources and exposure to risks, have remained unchanged. Or, as Mackenbach (2012:764) concludes, the FCT “stipulates that it is the social forces underlying social stratification that ultimately cause health inequalities, and not exposure to the proximal factors, which are usually studied by social epidemiologists.” Thus, the association between SES and health is reproduced over time even though specific disease profiles, risk factors, and protective resources may change (Link and Phelan 1995; Phelan et al. 2004, 2010). SES can therefore be conceptualized as “cause of causes” or the “risk of risks” (Phelan et al. 2010:S30).

### Approaches to Testing the Theory

Since its introduction, there have been several empirical tests of the FCT that have underpinned its importance and led to its widespread recognition in health sociology (for reviews, see Clouston and Link 2021; Phelan et al. 2010). Clouston and Link (2021) distinguish three approaches to the empirical testing of FCT that have been used in previous studies. First, the disease preventability approach examines the association between SES and mortality across multiple causes of death that differ in their preventability. According to the FCT, the associations of SES with mortality should be higher for more preventable diseases (e.g., lung or colorectal cancer) than for those that are less preventable (e.g., brain or ovarian cancer) because SES inequalities should be most apparent in cases where resources can be effectively deployed to gain a health advantage. This approach focuses on whether SES groups are affected differently by certain diseases depending on their preventability. The FCT would assume that people with a higher SES mainly die from less preventable diseases and that people with a lower SES die to a greater extent from more preventable diseases. Several studies support this assumption (e.g., Ghosn et al. 2017; Mackenbach et al. 2017; Masters et al. 2015; Phelan et al. 2004).

Second, in line with this reasoning, the preventability shifts approach focuses on the association between SES and mortality over time. In line with the FCT, individuals with high SES should be able to avoid premature death most effectively. As a result, SES inequalities in a given disease should emerge or increase the advantage of individuals with higher SES as new knowledge or technology to prevent the disease becomes available. Accordingly, this approach focuses on SES gradients in survival regardless of any specific health problem. The FCT would assume that a lower SES is associated with higher all-cause mortality compared to individuals with a higher SES, which is also supported by previous research (e.g., Rubin, Clouston, and Link 2014; Zapata-Moya, Willems, and Bracke 2019).

A third way of testing the FCT, the manipulated preventability approach, examines whether health inequalities occur in controlled trials where the intervention is randomly assigned. According to the FCT, individuals with a high SES can be expected to maximize their health benefits because their resources allow them to adopt the intervention more effectively than individuals with lower SES. Empirical support comes from studies showing that individuals with higher SES benefited more from health promotion interventions, such as breastfeeding promotion interventions (Yang et al. 2014), physical activity programs (Bann et al. 2016), and regeneration projects (Zapata-Moya and Navarro Yáñez 2017).

Despite the growing number of studies supporting the FCT, some findings contradict it, at least in part, leading some scholars emphasize the need to extend central features of the theory (for a review, see Clouston and Link 2021). One of the criticisms relates to counterintuitive findings regarding national differences in SES inequalities in health. Mackenbach, Kulhánová, Menvielle et al. (2015) and Mackenbach et al. (2017), among others, have shown that SES inequalities in mortality are not necessarily greater in economically more unequal countries. According to the authors, such heterogeneous findings may be due to “countervailing mechanisms as well as of other ‘meta-mechanisms’ than the theory implies” (Mackenbach et al. 2017:1130). For example, extensive welfare arrangements may have reduced social inequalities in some of the flexibly usable resources (e.g., income), limiting the causes of association between SES and mortality primarily to nonmaterial conditions, such as social or cultural capital (Mackenbach et al. 2017).

### Contexts with Largely Standardized Living Conditions: A Fourth Way to Test the FCT

As Phelan et al. (2004:269) put it, “[e]mpirically testing the importance of resources *per se* is difficult, because it requires the identification of situations in which the ability to use socioeconomic resources can be analytically separated from SES itself.” In what follows, we present a context in which the use of SES-related resources is separated (at least for the most part) from SES itself, so exactly what Phelan and colleagues have called for.

Our analyses are based on a unique data set containing the life data of Catholic monks from Germany born between 1840 and 1959, grouped into the six 20-years birth cohorts: 1840 to 1859, 1860 to 1879, 1880 to 1899, 1900 to 1919, 1920 to 1939, and 1940 to 1959. Monastic life serves as a quasi-experiment in the sense that it represents a social context in which all members have largely standardized living conditions regardless of the SES of the monks.

We analyze the mortality of the monks, distinguishing between “padres” (i.e., monks with university degrees who are predominantly in leading positions in the monastery) and “brothers” (i.e., monks without higher education and who typically work in a variety of occupations that would be considered lower SES in the general population). In the framework of the FCT, one might expect the influence of SES on mortality to be “muted” in this population because the link between SES and the flexibly usable resources that follow from it is removed. This study is not only unique in terms of the population studied but also in terms of the long time period covered. Between the birth cohorts 1840 to 1859 and 1940 to 1959, the spectrum of diseases and the associated mortality rates have changed tremendously, as have the decisive risk factors. The data thus allow us to test the temporal generality of our claim in the period under study.

### The Monastic Population in Health Research and Characteristics of the Monastic Life

Monastic populations have long intrigued health researchers and demographers for two main reasons. First, they represent a distinct group of individuals with typical, well-defined characteristics that distinguish them from the general population. Second, they have a long history of providing exceptionally reliable data because the life data of all members of the religious communities are carefully recorded and archived. The earliest study on life expectancy of order members dates back to the eighteenth century (Deparcieux 1746), documenting a higher life expectancy among French nuns and monks compared to the general population. Since then, the health and mortality of religious order members have become a recurring topic in demography and health research. Most studies focused on causes of death and life expectancy, until the seminal Nun Study (Snowdon 2001) provided unprecedentedly detailed medical and psychological examinations of nuns in the United States, providing new insights into the risk factors of Alzheimer’s disease. Overall, the health and mortality risks of cloistered populations are gender specific and partly change over time (see overviews in Flannelly et al. 2002; Luy 2003; Mackenbach et al. 1993). For example, in periods when infectious diseases, such as tuberculosis, were the major causes of death, nuns faced elevated mortality risks compared to the overall female population because many of them served as nurses in hospitals. The prevalence of other causes of death also varies, with higher breast cancer mortality among nuns but lower cervical cancer mortality compared to the general female population (overview in Luy 2003).

By contrast, relatively little is known about male order members. Some studies have documented a lower frequency of heart attacks among monks than in the general population (e.g. Groen et al. 1962), whereas others found no advantage for monks in terms of morbidity prevalence and other health indicators (Mackenbach et al. 1993). Based on data from Germany, it has been shown that monks have a considerably higher life expectancy than the general male population. In monastic life, the well-known gender gap in life expectancy is significantly reduced, with the maximum survival advantage for nuns being no more than one year in remaining life expectancy at young adult ages (Luy 2003). A recent study documents that the higher life expectancy of male monks is particularly due to a lower mortality rate among low-educated monks, whereas the life expectancy of highly educated monks was comparable to that of highly educated men in the general population (Luy, Wegner-Siegmundt, and Di Giulio 2021).

From a theoretical point of view, several features make the monastic life particularly interesting for health research, including in the context of the FCT. The monks, whose mortality is the subject of this study, lived in Catholic monasteries belonging to the Augustinian, Benedictine, Carmelite, and Cistercian orders, which practice a so-called “semi-contemplative” way of life. Unlike traditional “contemplative” orders, which live a life of silence and strict seclusion from the outside world, semi-contemplative orders still cultivate interaction with people outside the monastery. However, the living conditions and the way of life are characterized by a “simple lifestyle,” which is determined by vows of poverty, chastity, and obedience. Daily life follows a strict routine in terms of time for sleep, work, study, and recreation (see Klassen 2001).

Members of the order are typically not exposed to a range of health-related stressors: They are free from financial worries because the monastery provides lifelong shelter, and all members have identical living conditions, comparable food, and equal access to medical care. Because the monks live in chastity, they do not have to provide a family’s standard of living, and they do not experience marital conflict, worry about their offspring, or struggle to balance family and work life. The monastic life has another particular feature. Unlike single men in the general population, the monks’ status as “unmarried” does not mean that they cannot benefit from some of the health-protective resources typically associated with marriage. Social contacts within the monastery are close, and there is mutual help and care, a social network to provide support in the case of a critical life event, and social control. Monastic life thus offers some of the health-promoting factors associated with marriage for men (for more information on the lives of Catholic monks, see Luy 2003; Luy, Flandorfer, and Di Giulio 2015; Madigan and Vance 1957).

To put it in terms of the explanatory approaches to health inequalities, the monastic life provides a context in which material conditions (including housing) are standardized for all of its members. However, the monks do perform different kinds of work, ranging from leading positions and priests (predominantly padres) to secretaries, cooks, librarians, archivists, gardeners, or gatekeepers (predominantly brothers). Yet the typical occupations of monks do not include classic low-SES jobs with an extraordinary high risk of death or extremely unhealthy working conditions, such as work in industries with high exposure to toxic substances or coal mining. The monastic life also involves a certain standardization of health behaviors, although there are still some degrees of freedom. Similarities between monks, regardless of their SES, can also be assumed in terms of psychosocial resources. This applies at least to their integration into the community, the social contacts, and the mutual support and control that go along with it. Nevertheless, as in any other society, there are individual differences, for example, in coping styles and personality traits, that might be relevant for health.

In conclusion, the living conditions of this population are very similar to the situation called for by Phelan et al. (2004), where the ability to use SES-related resources can be analytically separated from SES itself. To our knowledge, this is the first study to use such an approach to test the FCT. We expect that in contrast to the general population, SES is not so closely linked to mortality among the monks because there are no longer (or at least much less) SES-related inequalities in the use of resources and the exposure to risks from the moment the monks enter the community. As mentioned previously, our data cover a long period of more than 150 years. We argue that the monastic life is a “comprehensive health protection” because it provides several beneficial living conditions, including not only material factors and working conditions but also several psychosocial resources and health behaviors. Thus, we expect a health-protective effect for monks with lower SES throughout the whole period.

## Data and Methods

### The German “Cloister Study”

We used data from the German “Cloister Study,” which contains life data of 5,781 Catholic monks born between 1588 and 1983 from four religious orders mainly located in southern Germany. The data were first collected in the years 1997 to 1998, including the Augustinians from Würzburg, the Carmelites from Bamberg, and the Missionary Benedictines from St. Ottilien. In 2006, the data were updated and extended to include in addition the Cistercians from Marienstatt located in western Germany. The main source for the data collection was the so-called “profession books” of the communities, in which each person who ever joined the community as a member is recorded with order title (padre, cleric, brother) and life data, that is, date of birth, date of entry into the order, and—if given—date of resignation or death. Some of the profession books contain further information, such as the monks’ missionary activities, education, occupation, and function(s) within the order. Unfortunately, the amount and completeness of this additional information varies between orders and communities and could not be used for the present analysis (for more details on data collection, see Luy 2003, 2009).

We restricted the analysis to the cohorts born between 1840 and 1959 and thus covering more than 150 years, resulting in a subsample of 2,857 Catholic monks. For the older cohorts, there were not enough members of the orders due to secularization at the time, and of the monks born since 1960, too few had died by the end of the data collection in 2006 to allow mortality analysis. Furthermore, we excluded monks dying or leaving the monastery below age 30 (n = 405) because of the starting age of our analysis and those with missing information on order titles (n = 31). These exclusions resulted in an analytic sample of 2,421 individuals.

### Order Titles as a Proxy of SES

Because the data sources from the monastery archives did not contain complete or consistently coded data on education and occupation, we approximated the SES from the monks’ order titles: padre, cleric, and brother. These order titles are closely linked to the education and occupation of the monks. The order title “padre” requires the completion of a university degree in theology. In terms of occupations, padres work as priests or pastors, and many of them occupy leading positions within the monastery. Monks with a degree who have not become priests are traditionally given the title of “cleric.” Due to their education, we assigned the clerics (n = 28 in our analysis sample) to the group of padres. By contrast, the order title “brother” is not directly linked to a specific educational degree. Most monks belonging to this group have a lower education level, as described, for instance, in Schwaiger’s (1994) encyclopedia of religious orders. Compared to padres, brothers work in more “simple” professional activities, for example, as secretaries, cooks, librarians, archivists, gardeners, or gatekeepers. In the following, we refer to padres (including clerics) as monks with high SES, and brothers will be referred to as monks with lower SES.

### Statistical Analysis

We estimated survivorship curves differentiating between monks with higher and lower SES using Kaplan-Meier product limit estimation from age 30. We took left truncation due to entries at higher ages and right censoring due to exits from the monasteries or monks being still alive at the time of data collection into account. Monks dying in combat during World War I or World War II (n = 105)—information included in the profession books—were treated as censored cases because the number of deaths in war were unevenly distributed among padres and brothers (25.7% padres, 74.3% brothers). To examine whether patterns of inequalities in mortality between monks with high and lower SES changed over time, we grouped the monks into the 20-year birth cohorts 1840 to 1859, 1860 to 1879, 1880 to 1899, 1900 to 1919, 1920 to 1939, and 1940 to 1959. The analyses were carried out separately for each birth cohort, so it is a longitudinal analysis of mortality over time for each cohort. The statistical significance of the differences in survival by SES was assessed using the log-rank test. The log-rank test is a large-sample chi-square test that provides an overall comparison of the Kaplan-Meier survival curves. The corresponding *p* value indicates whether the null hypothesis should be rejected (i.e., whether the survival curves are statistically equivalent; for details, see Kleinbaum 1996:58–62). All analyses were performed using the software package R, Version 3.0.1.

## Results

Table 1 presents descriptive statistics. The proportion of monks with lower SES ranged from 42.9% to 62.5 % across the birth cohorts. The number of person-years lived as a member of the order ranged from 2,095.2 years in the 1840 to 1859 cohort to 42,438.0 years in the 1900 to 1919 cohort. The latter also included the highest number of deaths (753 without war casualties), whereas the lowest number of monks died in the youngest cohort analyzed, 1940 to 1959 (18 deaths) because most were still alive at the time of data collection. The average age at entry into the convent varies across birth cohorts. In general, the average age at entry was higher for the monks with lower SES in all cohorts, with the differences tending to decrease from the older to the younger cohorts.

**Table 1.** Descriptive Characteristics of the Analyzed Sample of Catholic Monks Aged 30 Years and Older.

		Birth Cohorts					
		1840–1859	1860–1879	1880–1899	1900–1919	1920–1939	1940–1959
Individuals (%)	Lower SES	27 (46.6)	192 (62.5)	256 (56.5)	434 (48.8)	207 (42.9)	101 (43.9)
	High SES	31 (53.4)	115 (37.5)	197 (43.5)	456 (51.2)	276 (57.1)	129 (56.1)
Deaths (%)^a^	Lower SES	27 (49.1)	175 (63.8)	230 (54.5)	350 (46.5)	56 (45.2)	10 (55.6)
	High SES	28 (50.9)	107 (36.2)	192 (45.5)	403 (53.5)	68 (54.8)	8 (44.4)
Person-years	Lower SES	851.9	6,921.6	10,687.4	20,042.4	9,671.1	3,192.3
	High SES	1,243.2	4,228.4	9,031.4	22,395.5	12,175.3	4,144.8
Mean age at entry (*SD*)	Lower SES	32.9 (7.1)	28.0 (6.6)	26.2 (5.5)	22.3 (4.9)	22.9 (6.1)	23.8 (6.9)
	High SES	26.9 (7.8)	24.8 (6.8)	23.4 (5.5)	22.0 (5.4)	22.9 (6.1)	22.8 (4.3)
Mean age at death (*SD*)	Lower SES	61.9 (3.8)	65.6 (1.3)	71.3 (1.0)	75.1 (0.7)	77.9 (0.9)	63.9 (0.6)
	High SES	68.4 (2.7)	63.0 (1.5)	69.9 (1.1)	74.1 (0.7)	77.8 (0.8)	64.6 (0.5)

*Source*: German Cloister Study, own calculations.

*Note*: Case numbers for birth cohorts: 1840–1859, *n* = 58; 1860–1879, *n* = 307; 1880–1899, *n* = 453; 1990–1919, *n* = 890; 1920–1939, *n* = 483; 1940–1959, *n* = 230. SES = socioeconomic status; high SES = padres and clerics; low SES = brothers. SD = standard deviation.

a War casualties excluded (*n* = 105).

The mean ages at death and the corresponding standard deviations in the bottom row of Table 1 indicate that monks with high and lower SES experienced similar mortality. Each of the two SES groups had an advantage over the other in some of the six cohorts, with the differences ranging from a 6.5-year advantage for the monks with high SES in the 1840 to 1859 cohort to a 2.6-year advantage for the monks with lower SES in the 1860 to 1879 cohort. Note that the average age at death for the 1940 to 1959 cohort decreased relative to the 1920 to 1939 cohort because, as noted previously, most members of the youngest cohort were still alive and the earlier deaths necessarily occurred at younger ages.

**Figure 1.**
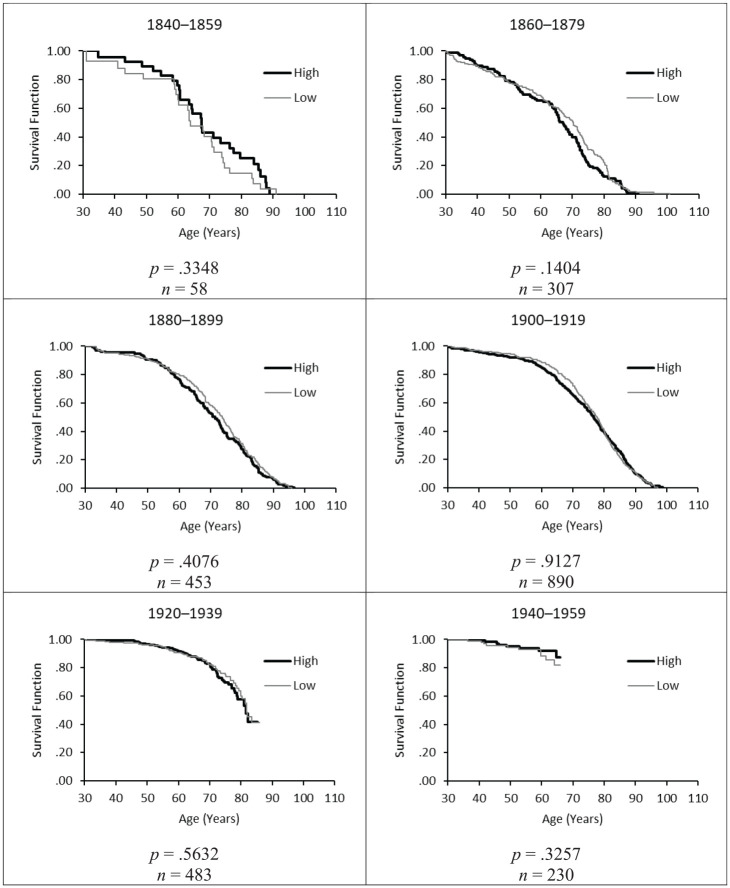
Kaplan-Meier Survivorship Curves from Age 30 for Catholic Monks by SES, Birth Cohorts 1840–1859 to 1940–1959. *Source*: German Cloister Study, own calculations. *Note*: War casualties are treated as censored cases at the time of death (n = 105). SES = socioeconomic status; high SES = padres and clerics; low SES = brothers.

The similar survival of Catholic monks with high and lower SES is further illustrated in Figure 1, which shows the Kaplan-Meier survivorship functions by SES for all cohorts analyzed. The survival curves for monks with high SES are displayed in bold black lines, and those of the monks with lower SES are shown in thin gray lines. In each cohort, the survival curves were crossing over several times. It appears that the monks with lower SES were somewhat disadvantaged in the younger adult ages between 30 and 50, whereas they tended to have slightly better survival in the older ages. Nevertheless, the difference in overall survival between the two groups of monks was not statistically significant in any of the cohorts (see the *p* values of log-rank tests in Figure 1), indicating that the null hypothesis that the survival curves of the two groups are statistically equivalent cannot be rejected.

## Discussion

### No Social Gradient in Mortality in a Population with Homogeneous Living Conditions

In this article, we used the special features of monastic life to test Link and Phelan’s (1995) fundamental cause theory (FCT). The study of Catholic monks has the great advantage that many factors related to SES differences in mortality in the general population (e.g., material living conditions and health behaviors) are directly controlled for due to the largely standardized living conditions in the monasteries. Furthermore, the long observation period across multiple birth cohorts allows us to test the temporal generality of one of the FCT principles: that SES associations with mortality persist regardless the disease patterns, causes of death, or main risk and protective factors in a given period. Other strengths include the high quality of the data in terms of completeness and accuracy of the life data and the fact that all analyses are based on longitudinal survival experience.

The results of this study provide additional support for the FCT in the sense that the SES-related inequalities in the access to resources and in exposure to risk factors appear to be “muted” under the conditions of the monastic life. Luy et al. (2021) compared the education-specific mortality of women and men from the general population and Catholic orders in the years 1984 to 1998. They found that the higher life expectancy of monks was mainly due to the low mortality of less educated monks, whereas the life expectancy of the highly educated in the general and monastic population was comparable. As the present study adds, the health-protective effect of monastic life for men with low SES can also be observed in a long historic period of time and is therefore independent of the changing spectrum of the causes of death and the associated risk factors.

Before discussing the implications of the results in more detail, the limitations of this study should be mentioned. Most importantly, the assignment of the SES of the monks based on the order titles allows only for a differentiation between the padres (and clerics) with high SES (academics who are often in leading positions within the monasteries) and brothers with lower SES. Due to the lack of information on the exact level of education and occupation, it was not possible to further differentiate within the groups of brothers between those with a medium SES and low SES. This would have provided an even more nuanced picture of the (absence of) the social gradient in mortality.

We would like to discuss some thoughts on the “selectivity” of the monastic population (i.e., potential health differences within and between the general and monastic population due to nonrandom processes). Theories of social selection assume processes of health selection into SES: More healthy and able individuals achieve a higher SES, whereas less healthy and less able individuals fail to move up or drift downward in SES. Consequently, the health of the padres (at least when entering the monastery) can be assumed to be better than that of the brothers. The available data do not allow us to test whether monks with high and lower SES differed in terms of baseline health. However, a number of health checks are carried out before admission to the monastery (Gajewski and Poznanska 2008; Luy 2003; Madigan 1957). People with serious—in some periods, even with mild—illnesses are not accepted into the monastery, so it is unlikely that the low-SES monks are characterized by a strong overall health disadvantage at the time of entry. Although this may imply positive selection depending on health at younger ages, the development of morbidity is a lifelong process, and studies suggest that social causation dominates over processes of social selection for explaining SES-related differences in the overall population, especially at older ages when serious health limitations and mortality increase (Hoffmann et al. 2018).

Another concern refers to processes of self-selection into the cloister life: It could be that people who decide for a life in the cloister differ from the general population with respect to unobserved characteristics, for example, spirituality and strength of will, which may, in turn, be associated with more beneficial health behaviors and consequently result in lower mortality. Although we cannot rule out these processes of self-selection, at the same time, we find no good argument or empirical evidence that the low-SES monks should be more selective than the higher educated ones. By contrast, a study on female members of religious orders from a study by Timio et al. (1999), which looked at medical conditions and health behaviors that could introduce bias when comparing the monastic and the general populations, found that nuns and the worldly women were similar in most of these conditions. More recent analyses of survey data on nuns and monks from Austria and Germany also found no evidence of strong selection effects in psychosocial characteristics, family background, and living conditions during childhood and adolescence that might bias our study (Bowen 2021; Krivanek and Wiedemann 2021). In summary, the decision to live in a monastery may seem extraordinary—but its specific characteristics are very likely to apply equivalently to all order members, no matter of an individual’s SES.

An important question that arises from our study is why exactly monks with low SES do not show a higher mortality compared to their counterparts with high SES. Theoretically, the equivalent survival experiences of padres and brothers might also result from an excessively high mortality of monks with high SES. This is, however, very unlikely. The study of Luy et al. (2021), who analyzed mortality differences by SES among Catholic order members in comparison to the general population for the period 1984 to 1998, shows that in particular, monks with low SES benefit from monastic life in terms of survival rates. Moreover, several studies on the general population have shown that adverse health behaviors are largely responsible for SES inequalities in mortality (e.g., Goldman 2001; Montez and Zajacova 2013). Given the standardizations in monastic life, it seems likely that monks with low SES do not adopt such unhealthy lifestyles. This effect might also be reinforced by what Freese and Lutfey (2011) would consider spillovers: Monks with higher SES could, by exerting social control over monks with lower SES, affect health behaviors in the overall population of order members in a positive way.

Because the spectrum of diseases and the associated mortality rates has changed, it is not enough to focus solely on SES-related inequalities in health behaviors, such as tobacco and alcohol consumption, because other risk factors may have been more important depending on the respective causes of death in a given period. In addition, we know that risky health behaviors often follow a process of social diffusion: Although the higher SES groups are often usually early adopters of behaviors such as smoking, they move away from these behaviors over time, whereas the lower SES groups maintain them (Link and Phelan 1995). In the context of our findings, this means that whatever the social distribution of risky health behaviors in the population and whatever the possible selection effects among monks, there were no SES-related differences in mortality among monks at any time.

Another likely health-protective factor for the monks with lower SES could be their working conditions. In the general population, studies show that occupational hazards can cause a number of serious diseases and injuries (Concha-Barrientos et al. 2004; Nurminen and Karjalainen 2001), accounting for about half of all occupational deaths and work-related illnesses, particularly in the transport and construction industries (Marsh et al. 2013). Typical hazardous occupations include agriculture, sewage treatment, waste collection, some manufacturing (e.g., vinyl chloride and chemical production), coal mining, and work involving exposure to asbestos (Concha-Barrientos et al. 2004). These occupations are not found among monks who mainly work in less risky, physically demanding, and stressful jobs (Luy 2003).

The social connectedness in the monastery may also contribute to the higher survival of the monks with lower SES. As Holt-Lundstad, Smith, and Layton (2010) conclude, the impact of (lacking) social relationships on mortality is comparable to other well-established risk factors for premature death, such as smoking. In the general population, SES affects people’s opportunities to engage in beneficial social contacts (Keim-Klärner et al. 2023). In this respect, too, monastic life, with its close ties between members, regular interactions, and the willingness to support each other, may be a safeguard for the health of monks with lower SES.

Finally, it might be assumed that monks are protected from “external” causes of death, such as accidents, poisoning, murder, suicide, and so on. Mortality from these causes of death is a factor contributing to lower life expectancy of men with lower SES in the general population. However, Luy (2009), using the same data set of Catholic monks, showed that mortality from external causes of death did not differ between monks and men from the general population. This can be interpreted as contradicting both the assumption that monks are particularly protected from external causes of death and the assumption that it is predominantly men with a low SES who do not tend to adopt harmful lifestyles (i.e., a highly selective group) choose to live in a religious community.

## Conclusions and Starting Points for Future Studies

The association between SES and mortality is very strong, typically amounting to a 5- to 10-year difference in average life expectancy at birth (Mackenbach 2012). However, under the conditions of monastic life, SES is not consistently associated with men’s mortality, suggesting that health-related benefits and harms are experienced more equally irrespective of people’s individual SES. In this way, our study underscores the importance of flexible resources, or rather, the lack thereof in the case of individuals with lower SES. As we have shown, with standardizing the living conditions that can be achieved with the resources that usually come with a higher SES, differences in health outcomes were largely eliminated to the benefit of people with lower SES.

It is beyond the scope of our study to test specific underlying mechanisms that may reduce the mortality risk of the monks with low SES because the data set does not include information on lifestyle, material living conditions, or psychosocial factors. To this end, in-depth analyses of the new detailed health survey of the German and Austrian monastic population that has been conducted within the Cloister Study will provide such insights in the future (for more information about the data, see Bowen and Luy 2016; Wiedemann et al. 2021). Another valuable avenue for future research could be to follow the presented new strategy to test the FCT by analyzing health inequalities in other “special” subpopulations with fairly homogeneous living conditions in which the ability to use socioeconomic resources can be analytically separated from SES itself.

The results of the presented study on Catholic monks provide additional and new support for the FCT. This is an important outcome for public health officials because the theory helps to better understand the causes of social inequalities in health and to find ways to alleviate them. Although the specific mechanisms underlying our findings are yet to be identified, the finding that monastic life appears to erase the social gradient in mortality, which is otherwise nothing less than a universal truth, is striking. A deeper understanding on the causes of the absence of a social gradient in health in a context, where social hierarchies are still evident, may help to develop strategies that enable societies to reduce the large and persistent disadvantages in health and mortality of underprivileged groups.
